# Cardiac Glycosides: From Natural Defense Molecules to Emerging Therapeutic Agents

**DOI:** 10.3390/biom15060885

**Published:** 2025-06-17

**Authors:** Arturo Ponce, Catalina Flores-Maldonado, Ruben G. Contreras

**Affiliations:** Department of Physiology, Biophysics and Neurosciences, CINVESTAV-Instituto Politecnico Nacional, Mexico City 07360, Mexico; catalina.flores@cinvestav.mx (C.F.-M.); gerardo.contreras@cinvestav.mx (R.G.C.)

**Keywords:** cardiac glycosides, Na^+^/K^+^-ATPase, signal transduction pathways, cancer therapy, antiviral agents, drug repurposing

## Abstract

Cardiac glycosides (CGs), a class of plant- and animal-derived compounds historically used to treat heart failure, have garnered renewed interest for their diverse pharmacological properties beyond Na^+^/K^+^-ATPase (NKA) inhibition. Recent studies reveal that CGs modulate key signaling pathways—such as NF-κB, PI3K/Akt, JAK/STAT, and MAPK—affecting processes central to cancer, viral infections, immune regulation, and neurodegeneration. In cancer, CGs induce multiple forms of regulated cell death, including apoptosis, ferroptosis, pyroptosis, and immunogenic cell death, while also inhibiting angiogenesis, epithelial–mesenchymal transition, and cell cycle progression. They demonstrate broad-spectrum antiviral activity by disrupting viral entry, replication, and mRNA processing in viruses such as HSV, HIV, influenza, and SARS-CoV-2. Immunologically, CGs regulate Th17 differentiation via RORγ signaling, although both inhibitory and agonistic effects have been reported. In the nervous system, CGs modulate neuroinflammation, support synaptic plasticity, and improve cognitive function in models of Alzheimer’s disease, epilepsy, and multiple sclerosis. Despite their therapeutic potential, clinical translation is hindered by narrow therapeutic indices and systemic toxicity. Advances in drug design and nanocarrier-based delivery are critical to unlocking CGs’ full potential as multi-target agents for complex diseases. This review synthesizes the current knowledge on the emerging roles of CGs and highlights strategies for their safe and effective repurposing.

## 1. Introduction

Cardiac glycosides (CGs) are a structurally diverse class of naturally derived compounds. Found as secondary metabolites in various flowering plants, they are also produced by certain animals as a means of predator deterrence. The term *cardiac glycosides* stems from their characteristic chemical framework and their historically established use as cardiotonic drugs [1,2].

Throughout history, humans have recognized both the medicinal and toxic properties of CG-producing organisms. Some species were avoided due to their harmful effects, while others were deliberately used as poisons in hunting and warfare. CGs also found applications in traditional medicine. For example, the ancient Egyptians extracted CGs from *Nerium oleander* to treat heart conditions [3], while *Digitalis purpurea* (foxglove) was historically administered to manage edema. Traditional Chinese medicine likewise incorporates CGs; *Chansu*—derived from toad skin and venom—has been used for centuries to treat inflammatory disorders and certain cancers [4,5].

The cardiotonic properties of CGs were first scientifically documented in the late 18th century by the English physician William Withering, who demonstrated the efficacy of digitalis in treating dropsy by improving and regulating cardiac function [6]. Subsequent research led to the isolation of key active components, such as digoxin and digitoxin [7]. It was later discovered that CGs exert their cardiotonic effects through partial inhibition of the Na^+^/K^+^-ATPase (NKA) pump, resulting in increased intracellular calcium and enhanced myocardial contractility [8]. However, due to their narrow therapeutic window, many CGs have been replaced by safer alternatives—though digoxin remains in clinical use as the primary cardiotonic agent [9].

Despite their toxicity, CGs have regained attention for their selective cytotoxicity toward stressed or malignant cells, making them promising candidates for cancer therapy [10] and antiviral treatment [11]. Emerging evidence also suggests potential neuroprotective effects, further broadening their therapeutic relevance [12]. Mechanistically, NKA functions not only as an electrogenic pump that maintains ion homeostasis but also as a key membrane receptor involved in signal transduction [13]. CG binding induces conformational changes in NKA that activate signaling pathways such as Src/Ras/Raf/MEK/ERK, PI3K/Akt/mTOR, NF-κB, and PKC-dependent cascades [14]. These pathways regulate essential cellular functions including proliferation, differentiation, resistance to apoptosis, and inflammatory responses, implicating CGs in both physiological and pathological processes.

Recent research has substantially deepened our understanding of CGs. Novel types and producer species have been identified, and advanced techniques—such as molecular docking simulations and crystallography—have clarified the molecular basis of CG–NKA interactions. Additional molecular targets beyond NKA are also being explored. Efforts to overcome CGs’ narrow therapeutic index—such as the development of synthetic derivatives and targeted delivery methods—have further expanded their clinical potential. Moreover, co-evolutionary studies have illuminated the ecological drivers behind the structural diversity of CGs in plants.

This review provides a comprehensive examination of the historical significance and pharmacological activities of cardiac glycosides, explores recent molecular insights, and highlights their emerging roles in oncology, immunotherapy, antiviral therapy, and other fields.

## 2. Chemical Diversity and Origin of Cardiac Glycosides

**Figure 1:**
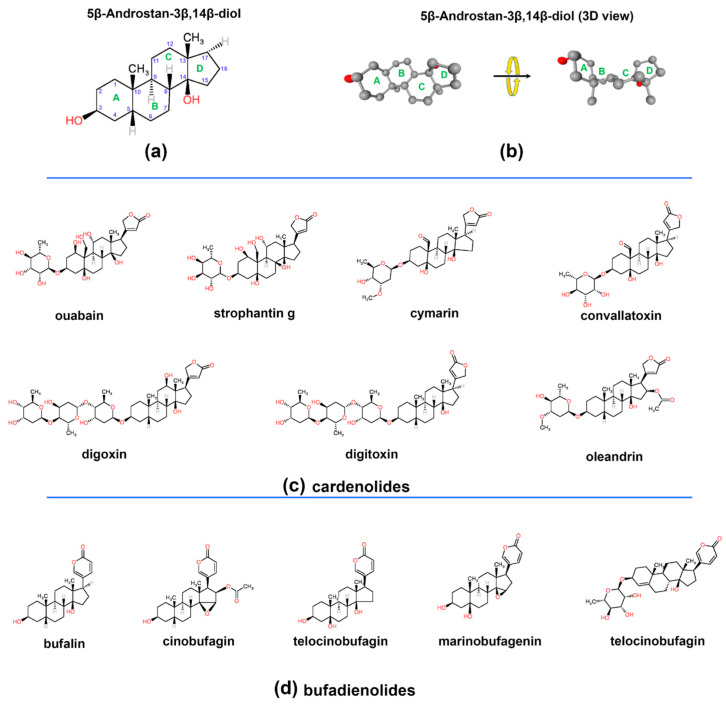
Chemical structure and diversity of cardiac glycosides. (**a**) Core steroidal structure common to all CGs, showing the carbon numbering across the four fused rings (A, B, C, and D). (**b**) Three-dimensional representation of the steroidal nucleus, highlighting the characteristic U-shaped conformation of the pharmacophore. (**c**) Chemical structures of representative cardenolides. (**d**) Chemical structures of representative bufadienolides.

The chemical structure of cardiac glycosides (CGs) comprises a steroidal aglycone core—specifically, a 5β,14β-androstane-3β,14-diol (Figure 1a)—linked to a sugar moiety at the C-3β position and a lactone ring at the C-17β position [15]. A defining feature of CGs is their characteristic “U”-shaped pharmacophore, formed by the cis–trans–cis fusion of the A/B, B/C, and C/D rings, respectively (Figure 1b). This conformation is essential for their interaction with Na^+^/K^+^-ATPase (NKA) and distinguishes them from other steroidal compounds [16]. While the steroid nucleus primarily confers pharmacological activity, the glycoside moiety modulates solubility, absorption, and overall bioavailability.

CGs are broadly classified into two major subtypes based on the structure of the lactone ring: cardenolides and bufadienolides. Cardenolides possess a five-membered α,β-unsaturated γ-lactone ring, whereas bufadienolides contain a six-membered α-pyrone ring [17]. This structural difference significantly influences their pharmacodynamics and toxicity. Bufadienolides typically exhibit higher potency and a narrower therapeutic index due to their stronger inhibition of NKA, which elevates the risk of cardiotoxicity [18]. In contrast, cardenolides—though still potent—generally present a more favorable therapeutic window and have been widely used in clinical settings to treat heart failure and atrial fibrillation [19]. Representative chemical structures of cardenolides and bufadienolides are depicted in Figure 1c and Figure 1d, respectively.

### 2.1. Cardenolides

Cardenolides are predominantly synthesized by plants as chemical defenses against herbivores. These compounds have been identified in over 17 plant families, encompassing hundreds of species across approximately 70–80 genera [20,21]. A recent review documented 295 cardenolides isolated from more than 30 higher plant species between 2010 and 2023 [22]. Among these, three novel cardenolides—castheveside A, castheveside B, and 3α-thevetiogenin—were recently isolated from *Cascabela thevetia* fruits [23].

The most prominent plant families and genera known to produce cardenolides include:

- Apocynaceae: *Asclepias*, *Nerium*, *Thevetia*, *Strophanthus*, *Cerbera*, *Calotropis* (over 100 cardenolides identified).
- Plantaginaceae: *Digitalis.*
- Brassicaceae: *Erysimum.*
- Ranunculaceae: *Adonis.*
- Hyacinthaceae: *Ornithogalum.*
- Moraceae: *Antiaris.*
- Euphorbiaceae: *Euphorbia.*
- Fabaceae: *Corchorus.*

Well-known cardenolide-producing species include *Digitalis purpurea* and *Digitalis lanata* (foxglove), *Nerium oleander* (oleander) [24,25], *Thevetia peruviana* (yellow oleander), *Strophanthus gratus* and *S. kombe*, *Calotropis procera* and *C. gigantea* (milkweeds) [26], *Asclepias curassavica*, *Drimia maritima* (Mediterranean squill), *Convallaria majalis* (lily of the valley), and *Adonis vernalis* (pheasant’s eye). Key cardenolides derived from these species include digoxin and digitoxin (*Digitalis* spp.), ouabain (*Strophanthus* spp.), convallatoxin (*Convallaria majalis*), calotropin and uscharin (*Calotropis* spp.), and thevetin (*Thevetia peruviana*). While these compounds have historically been employed to treat heart conditions, improper use can result in toxicity in both humans and animals.

**Figure 2:**
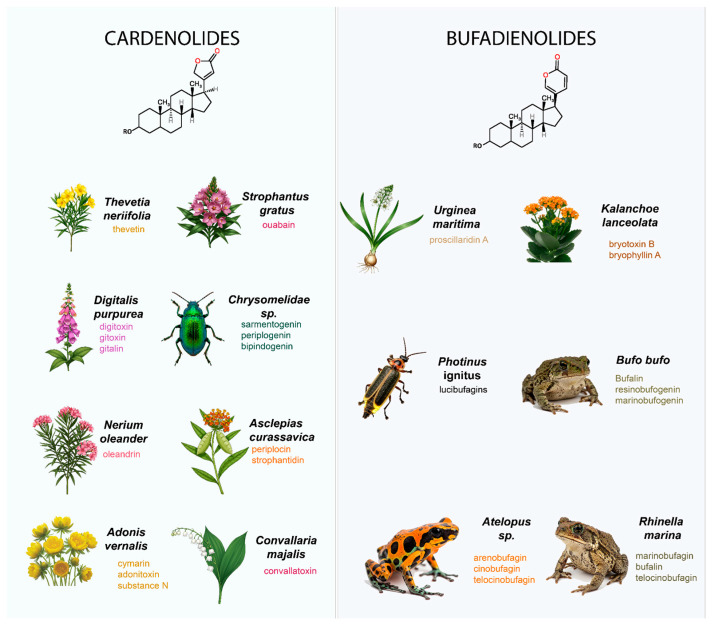
Diversity and origin of naturally occurring cardiac glycosides (CGs). The top panel illustrates the structural differences between cardenolides and bufadienolides. The lower panels depict various plant and animal species known to produce CGs—cardenolide-producing species are shown on the left, and bufadienolide-producing species on the right. For each organism, both the scientific name and the corresponding type(s) of CGs they produce are indicated.

Although plants are the primary producers of cardenolides, certain animal species are also capable of synthesizing these compounds. Notably, beetles in the family Chrysomelidae produce cardenolides such as sarmentogenin, periplogenin, and bipindogenin. Figure 2 (left) shows several plant and animal species that produce cardenolides, along with the names of the corresponding cardiac glycosides (CGs) they synthesize.

### 2.2. Bufadienolides

Bufadienolides are characterized by a six-membered α-pyrone (lactone) ring attached at the C-17β position of the steroidal (5β,14β-androstane-3β,14-diol) core [27]. The term “bufadienolide” originates from *Bufo* toads, whose venom contains these potent steroidal compounds. Historically, bufadienolide-containing plants were used as early as ancient Egypt, where squill (*Drimia maritima*, family Hyacinthaceae) was employed to treat heart disease [28]. The first bufadienolide glycoside to be structurally characterized was Scillaren A, identified in 1933 [29].

Beyond plants, bufadienolides are also produced by several animals, particularly toads of the Bufonidae family. The venom of approximately ten Bufo species has been confirmed to contain bufadienolides and their esters [30]. Traditional Asian medicines such as Ch’an Su and Senso are prepared from toad venom rich in these compounds [5]. Other amphibians, such as bufonid frogs of the genus *Atelopus*, produce related bufadienolides like telocinobufagin and bufotalin. Interestingly, lucibufagins—a structurally related subclass of bufadienolides—have also been identified in fireflies (*Photinus ignitus* and *P. marginellus*), where they serve as chemical deterrents against predators.

Among plants, bufadienolides have been reported in six families: Crassulaceae, Hyacinthaceae, Iridaceae, Melianthaceae, Ranunculaceae, and Santalaceae. The genus *Kalanchoe* (Hyacinthaceae) is particularly noteworthy for its high bufadienolide content [31]. In recent years, several studies have described the remarkable structural diversity of bufadienolides in *Helleborus* spp. [32,33], some of which exhibit potent cytotoxic effects against breast and cervical cancer cells [34].

Due to their cytotoxic and anticancer activities, bufadienolides are considered promising candidates for drug development, underscoring the need for further pharmacological and clinical investigation [35]. Among them, bufalin is one of the most extensively studied, having demonstrated anticancer [36], anti-inflammatory [37,38], and antiviral properties [39].

Figure 2 (right) depicts several bufadienolide-producing species, both animal and plant, along with the names of the corresponding cardiac glycosides (CGs) they synthesize.

### 2.3. Endogenous CGs

Although still debated, accumulating evidence suggests that mammals—including humans—endogenously produce compounds that are structurally and functionally analogous to cardiac glycosides (CGs). Due to their physiological relevance, these substances have been proposed as a novel class of steroid hormones [40]. A defining feature of CGs is their high-affinity binding to and inhibition of the sodium–potassium ATPase (NKA), which led researchers to hypothesize the existence of endogenous counterparts to plant- and animal-derived CGs [41]. This search was further motivated by efforts to identify a “hypothetical natriuretic hormone” involved in promoting sodium excretion and regulating blood pressure and vascular tone [42].

Between 1989 and 1991, a compound indistinguishable from ouabain—termed endogenous ouabain (EO)—was identified in human plasma [43]. EO is synthesized in the adrenal cortex, with its secretion regulated by epinephrine and angiotensin II [44]. Other endogenous cardiotonic steroids (ECTS), including EO, are also produced in the hypothalamus and are regulated by adrenocorticotropic hormone (ACTH), α-adrenergic and dopaminergic stimuli, angiotensin II (via AT2 receptor activation), hypoxia, and physical activity [40].

Elevated EO levels have been associated with hypertension, kidney dysfunction, and heart failure in both humans and animal models [45]. Additional endogenous CG-like compounds include digoxin, marinobufagenin [46], telocinobufagin [47], bufalin, 19-norbufalin, and proscillaridin A [46,47,48,49].

Despite their different origins, endogenous and exogenous CGs share several notable similarities, including a conserved steroidal structure, a lactone ring, and a high binding affinity for NKA. Both classes can inhibit the pump’s ion-transport function and initiate downstream signaling cascades. However, endogenous CGs are typically produced at nanomolar to picomolar concentrations and are thought to play physiological roles in maintaining sodium balance, vascular tone, and neuroendocrine regulation. In contrast, exogenous CGs—such as digoxin, ouabain, and bufalin—are administered at pharmacological doses and are primarily used to treat heart failure and arrhythmias.

Because both endogenous and exogenous CGs target overlapping NKA binding sites, competitive or synergistic interactions are possible. In pathological conditions characterized by elevated endogenous CG levels—such as heart failure or preeclampsia—these interactions may influence tissue responsiveness to therapeutic CGs, potentially enhancing efficacy or increasing toxicity. Therefore, understanding the interplay between endogenous and exogenous CGs is essential for optimizing treatment strategies and minimizing adverse effects, particularly in patients with altered endogenous CG profiles.

### 2.4. CG’s Derivatives

**Figure 3:**
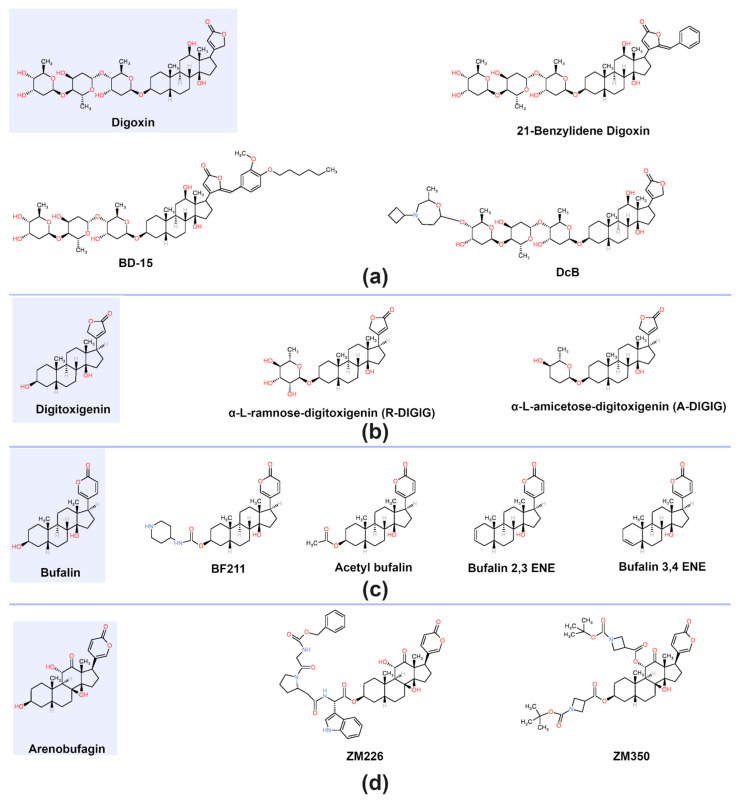
Newly developed cardiac glycoside (CG) derivatives. Panels (**a**–**d**) show the chemical structures of digoxin, digitoxin, bufalin, and arenobufagin, respectively, along with their corresponding derivatives. In each panel, the chemical structure of the parent CG is highlighted in violet.

In recent years, several chemically modified derivatives of natural cardiac glycosides (CGs) have been developed to improve their physicochemical properties, selectivity, and therapeutic efficacy. Figure 3 illustrates the chemical structures of parent CGs and representative derivatives, which are described below.

#### 2.4.1. Digoxin and Digitoxigenin Derivatives

Rocha and colleagues synthesized 21-Benzylidene Digoxin (21-BD) by introducing a styrene group into the lactone ring of digoxin—a modification shown to enhance biological activity [50]. Parreira et al. developed BD-15, a semi-synthetic γ-benzylidene derivative of digoxin that selectively enhances α3-NKA activity in the rat hippocampus and prefrontal cortex, with minimal impact on cardiac function [51]. Barathi et al. reported that DcB, a cyclobutyl derivative of digoxin, selectively inhibits the α2 isoform of NKA and effectively reduces intraocular pressure in ocular hypertensive nonhuman primates [52]. Additionally, O’Doherty’s group synthesized two digitoxigenin derivatives by modifying the sugar moiety with rhamnose or amicetose. Rhamnose substitution increased NKA affinity by 5–15-fold, while amicetose had no significant effect. Both derivatives elevated H_2_O_2_ levels, induced membrane lipid peroxidation, and reduced intracellular glutathione (GSH) levels [53].

#### 2.4.2. Bufalin Derivatives

Lei et al. synthesized BF211, a bufalin derivative featuring a carbamate group at the C3 position. BF211 demonstrated stronger pro-apoptotic effects and lower toxicity compared to native bufalin [54]. Acetylation at the same position produced acetyl-bufalin, which exhibited enhanced antitumor activity against non-small-cell lung cancer [55]. Sampath and colleagues synthesized bufalin 2,3-ene and bufalin 3,4-ene, both of which retained the biological activity of bufalin while demonstrating reduced cytotoxicity [56].

#### 2.4.3. Arenobufagin Derivatives

Chen and co-workers (2021) developed ZM226, a peptide-substituted arenobufagin derivative that showed enhanced antitumor activity and reduced cardiotoxicity [57]. Similarly, Tang et al. synthesized arenobufagin derivatives containing 3,11-bispeptide esters. Among these, ZM350 significantly inhibited tumor growth by 58.8% in an A549 nude mouse model, while maintaining low cardiac toxicity [58].

Overall, these chemically engineered CG derivatives demonstrate improved therapeutic indices, enhanced selectivity, and reduced systemic toxicity. Their development highlights the potential of CG analogs as next-generation therapeutic agents in both cancer and non-cancer applications.

## 3. Na^+^/K^+^-ATPase, a Pump and a Receptor of CGs

Since its discovery by Jens Chr. Skou over five decades ago [59], Na^+^/K^+^-ATPase (NKA) has been closely associated with our understanding of cardiac glycoside (CG) pharmacology. Although both the conceptual framework of NKA function and CG pharmacology have evolved over time, their interconnection remains robust. Until recently, it was widely accepted that NKA was the sole molecular target of CGs and that all their known effects—both therapeutic and toxic—were mediated through this enzyme. While a few exceptions have emerged, this view remains largely valid. More recently, however, NKA has been recognized not only as an electrogenic ion pump but also as a membrane receptor. Upon binding with exogenous or endogenous CGs, NKA can activate diverse intracellular signaling pathways involved in both physiological regulation and pathogenesis.

Although several comprehensive reviews have discussed recent advances in the structural and functional understanding of NKA (e.g., Contreras et al., 2024 [13]), the following subsections provide a focused overview of its core characteristics, emphasizing how CGs influence NKA function—not only as an ion pump and signaling receptor but also in its lesser-known role as a cell adhesion molecule.

### 3.1. NKA Molecular Structure and Diversity

**Figure 4:**
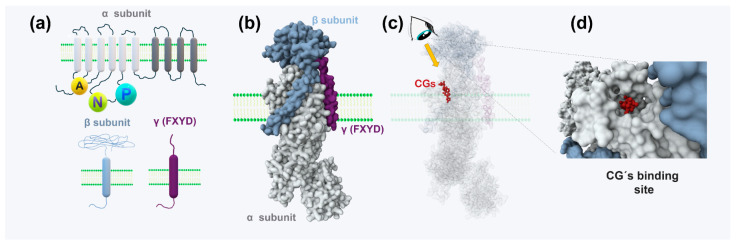
NKA, a pump and receptor of cardiac glycosides (CGs). (**a**) Topological profile of the constituent subunits. (**b**) Three-dimensional representation of the molecular surface of an NKA unit seen in profile, embedded in the membrane. (**c**) Same representation as in (**b**) but in molecular volume, to highlight the position occupied by a CG molecule. (**d**) Molecular surface view from the perspective indicated by the eye and arrow in scheme (**c**), highlighting the CG binding site. The images in (**b**–**d**) were created in RCSB PDB (http://www.rcsb.org/) (accessed on 15 February 2025) [70] with the molecular graphics program Mol* [71] from PDB ID 7WYT [72].

NKA is a heteromeric protein complex composed of three subunits: α, β, and γ (also known as FXYD) (Figure 4a,b). The α-subunit (~110 kDa) spans the membrane ten times and contains cytoplasmic N-, P-, and A-domains critical for ATP hydrolysis and ion transport. Its extracellular loops contribute to ion selectivity and interact with the β-subunit. Four α isoforms exist in mammals: α1 is ubiquitous and abundant in the kidney; α2 is expressed in muscle, heart, and brain; α3 is neuron-specific and essential for synaptic transmission; and α4 is testis-specific and crucial for sperm motility [60,61].

The β-subunit is a single-pass type II transmembrane protein with a short cytoplasmic N-terminus, a transmembrane domain, and a large extracellular region stabilized by disulfide bridges. It plays key roles in NKA assembly, membrane localization, and enzymatic stability [62]. Three β isoforms exist: β1 is ubiquitous; β2 (also known as AMOG) is enriched in glial and epithelial cells; and β3 is expressed in muscle, neurons, and other specialized tissues [63].

FXYD proteins are small, single-span transmembrane modulators that associate with the α-subunit to fine-tune pump activity in response to physiological factors such as ion concentrations, pH, and hormonal signals.

### 3.2. CG’s Influence on NKA Functions

#### 3.2.1. CGs Inhibit NKA as a Pump

The primary role of NKA is to actively transport sodium (Na^+^) and potassium (K^+^) ions across the cell membrane against their concentration gradients, using energy from ATP hydrolysis. The α-subunit serves as the catalytic core, binding ATP, Na^+^, and K^+^, while the β-subunit ensures proper assembly and membrane localization. FXYD proteins dynamically regulate activity in response to metabolic and environmental cues.

CGs inhibit NKA by binding to a specific pocket on the extracellular domain of the α-subunit during the K^+^ binding and dephosphorylation stage of the pump cycle (Figure 4c,d). At this point, the pump adopts the E2-P conformation, having expelled Na^+^ and awaiting K^+^ binding. CG binding prevents dephosphorylation, locking the pump in an inactive state [64].

Recent cryo-electron microscopy (cryo-EM) and molecular docking studies have mapped the cardiotonic steroid (CTS) binding site in detail. Key interactions occur between CGs and extracellular loops and transmembrane domains (particularly M1–M6 and M9–M10). The steroid nucleus inserts into a hydrophobic cavity, the lactone ring at C-17 forms stabilizing interactions, and the sugar moiety at C-3β enhances affinity via hydrogen bonding [65]. The depth of steroid core insertion is critical for determining the mode and efficacy of inhibition [19,66].

#### 3.2.2. NKA’s Role as a Signal-Transducing Receptor Activated by CG’s Binding

More than two decades ago, it was proposed that NKA could function as a receptor for ouabain and other CGs. This was later confirmed, especially for a subset of non-ion-transporting NKA molecules localized in caveolae—specialized membrane microdomains—where they form multiprotein complexes known as “signalosomes” [67]. Upon CG binding, NKA interacts with nearby signaling proteins, triggering intracellular pathways that regulate proliferation, differentiation, and apoptosis [68,69].

**Figure 5:**
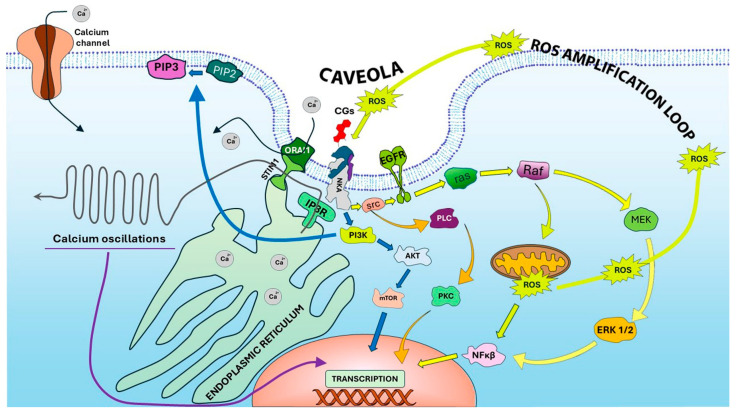
Signaling pathways activated by the binding of CGs to NKA. Arrow colors correspond to different signaling pathways, as detailed in the text.

The following subsections summarize the main CG-induced signaling pathways (see Figure 5).

##### Signaling Pathways Activated by CG Binding to NKA

Therapeutic doses of CGs can induce cardiac hypertrophy, suggesting a role in promoting growth beyond ion transport inhibition. Xie and Askari showed that ouabain induces immediate-early genes like c-fos, c-jun, and AP-1 in cardiac myocytes [73]. Ouabain activates Src kinase, which transactivates the epidermal growth factor receptor (EGFR), triggering the Ras/Raf/MEK/ERK cascade (Figure 5, yellow arrows) [74].

The partial inhibition of ouabain-induced ERK signaling by protein kinase C (PKC) inhibitors implicated additional pathways, including PLC/PKC signaling and Ca^2+^-dependent mechanisms (Figure 5, orange arrows) [75]. Moreover, ouabain enhances mitochondrial ROS production, activating MAPKs via Src-independent mechanisms [76,77]. CGs also stimulate PI3K/Akt/mTOR signaling independently of Src (Figure 5, blue arrows) [78].

Xie’s group showed that NKA, Src, and EGFR co-localize in caveolae and that NKA contains conserved caveolin-binding motifs, supporting its role as a scaffold in multiprotein signaling complexes [68].

##### CG-Induced Ca^2+^ Oscillations

In epithelial cells, ouabain induces intracellular calcium oscillations through a mechanism involving the inositol 1,4,5-trisphosphate receptor (IP3R) [79]. This activation does not rely on PLC-generated IP3 but rather on a direct interaction between NKA and IP3R within a complex stabilized by ankyrin B [77]. The LKK motif in the N-terminal domain of the NKA α-subunit is essential for IP3R binding [80].

This Ca^2+^ signaling promotes proliferation, adhesion, and apoptosis resistance via NF-κB activation. Phosphoproteomic analyses also reveal CAMK2G activation after ouabain stimulation [81].

##### CG-Induced ROS-Mediated Signaling

Reactive oxygen species (ROS) play a central role in CG-induced signaling through NKA. Molecules such as superoxide anion, hydrogen peroxide, and hydroxyl radicals act not only as metabolic by-products but also as secondary messengers in several NKA-mediated pathways [82]. Initial evidence linked ROS production to the activation of mitochondrial pathways downstream of Src activation (Figure 5, lime green path) [77]. Furthermore, antioxidant treatments were shown to partially inhibit ouabain-induced activation of MAPK, NF-κB, and protein synthesis, indicating that ROS are involved in both the catalytic and non-catalytic signaling effects of CGs [83].

##### Gene Regulation by CG-Induced Shifts in [Na^+^]_i_/[K^+^]_i_ Ratios

CG-induced shifts in the intracellular sodium-to-potassium ratio can directly influence gene expression [84]. In endothelial cells, elevated extracellular Na^+^ modulates genes linked to vascular function [85]. Prolonged CG exposure alters hundreds of transcripts, as seen in HUVECs treated with ouabain or marinobufagenin [86]. One proposed mechanism involves monovalent cation sensors, such as DNA G-quadruplexes, that regulate transcription in response to ionic changes [87].

#### 3.2.3. NKA’s Role in Cell–Cell Adhesion

Beyond its pumping and signaling roles, NKA contributes to intercellular adhesion in epithelial tissues [88]. In these cells, basolaterally localized β_1_ subunits from adjacent cells engage in direct interactions [89,90]. Fluorescence resonance energy transfer (FRET) studies confirm β_1_–β_1_ interactions, particularly at N-glycan-containing regions spanning residues 221–229 and 198–207 [91,92]. Structural alterations to β_1_ impair junction stability [93].

NKA also interacts with E-cadherin via the β_1_ subunit, reinforcing adherens junctions and epithelial cohesion [94]. In the retinal pigment epithelium (RPE), NKA localizes apically due to β_2_-mediated targeting [95]. Ouabain enhances β_1_–β_1_ adhesion via a Src-dependent pathway [96].

In the nervous system, the β_2_ subunit (AMOG) promotes neuron–glia adhesion [97,98]. Though heterologous expression suggests β_2_–β_2_ interactions, in vivo confirmation is pending [99]. Notably, astrocytes do not adhere to each other via AMOG, indicating that β_2_-mediated adhesion is selective for neuron–glia interactions [100].

These findings underscore NKA’s multifunctionality in epithelial and neuronal tissue architecture, influenced by β isoform composition and CG exposure.

## 4. Cardiac Glycosides: Modulators of Diverse Signaling Pathways

Recent advances reveal the remarkable diversity by which cardiac glycosides (CGs) modulate cellular signaling pathways. Their effects—either activating or inhibiting specific intracellular cascades—are highly dependent on cell type, tissue context, and physiological state. This complexity positions CGs as versatile modulators of critical biological processes such as proliferation, apoptosis, inflammation, and differentiation. These actions are shaped by context-dependent interactions within complex signaling networks.

The following subsections concisely overview major CG-influenced pathways, highlighting their biological significance and recent discoveries on specific CG regulation, offering insights into the therapeutic potential for cancer, fibrosis, and immune modulation.

### 4.1. PI3K/Akt Pathway

The phosphoinositide 3-kinase/protein kinase B (PI3K/Akt) pathway is central to the regulation of cell growth, survival, metabolism, and proliferation. Dysregulation of this pathway is implicated in cancer, diabetes, and neurodegenerative diseases. Various CGs modulate PI3K/Akt signaling, affecting apoptosis, survival, and metastasis:

- Cerberin inhibits PI3K/Akt/mTOR signaling in cancer [101].
- Bufalin suppresses gastric cancer progression [102] and hepatoma invasion [14].
- Strophanthidin and Lanatoside C attenuate PI3K/Akt/mTOR, reducing tumor growth and metastasis [103].
- Oleandrin inhibits LRP4/MAPK/NF-κB, preventing osteoclast differentiation [104].

### 4.2. TGF-β/Smad Pathway

The transforming growth factor-beta (TGF-β)/Smad pathway governs cell proliferation, differentiation, and immune regulation. Its dysregulation contributes to cancer progression, fibrotic diseases, and immune disorders.

- Digoxin inhibits TGF-β1/Smad signaling, preventing fibroblast differentiation into cancer-associated fibroblasts (CAFs) (CAFs) [105].
- Periplocymarin activates TGF-β/Smad signaling, protecting against myocardial fibrosis [106].

### 4.3. HIF-1α Signaling

Hypoxia-inducible factor 1 (HIF-1), particularly its oxygen-sensitive subunit HIF-1α, orchestrates cellular responses to hypoxia by regulating genes involved in angiogenesis, metabolism, and survival [107]. Several CGs have been shown to inhibit HIF-1α signaling:

- Digoxin, ouabain, and proscillaridin A inhibit HIF-1α protein synthesis and target gene expression [108].
- Digoxin suppresses hypoxia-induced VEGF and NDRG1 expression [109].
- Digitoxin inhibits HIF-1α and STAT3 in KRAS-mutant colon cancer [110].
- Bufalin enhances photodynamic therapy by inhibiting SRC-3/HIF-1α [110] and targets mTOR/HIF-1α in ovarian carcinoma [111].
- Cardenolides from *Calotropis gigantea* inhibit HIF-1 transcriptional activity [112].

### 4.4. JAK/STAT Pathway

The Janus kinase/signal transducer and activator of transcription (JAK/STAT) pathway mediates responses to cytokines and growth factors, playing essential roles in cell proliferation, immune regulation, and inflammation. Several CGs modulate this pathway in disease-specific contexts:

- Periplogenin inhibits JAK2/3-STAT3 signaling to reduce synovial proliferation in arthritis [113].
- Bufalin suppresses JAK/STAT to reduce inflammation in cancer and cardiovascular diseases [38].
- Periplocymarin alleviates cardiac hypertrophy via JAK2/STAT3 inhibition [114].
- Bufothionine induces autophagy in hepatoma-bearing mice through JAK2/STAT3 blockade [115].
- Convallatoxin inhibits colorectal cancer proliferation via JAK2/STAT3 and mTOR/STAT3 pathways [116].
- Peruvoside also targets PI3K/Akt/mTOR in cancer cells [117].

### 4.5. PERK/elF2α/ATF4/CHOP Pathway

This signaling axis is a major component of the unfolded protein response (UPR) triggered by endoplasmic reticulum (ER) stress, determining cell fate through adaptation or apoptosis.

- Oleandrin activates the PERK/eIF2α/ATF4/CHOP pathway, inducing immunogenic death in breast cancer cells [118].
- Neriifolin induces ER stress-mediated apoptosis in prostate cancer by activating PERK and CHOP, impairing DNA repair mechanisms [119].

As demonstrated, CGs exhibit complex and context-dependent modulation of key signaling pathways—including PI3K/Akt, TGF-β/Smad, HIF-1α, JAK/STAT, and UPR—across diverse pathological states. This multifaceted interaction underpins their significant therapeutic potential in cancer (inhibiting growth, survival, metastasis), fibrosis (modulating TGF-β), inflammation (targeting NF-κB, JAK/STAT), and immune regulation. Future research should focus on elucidating the precise context-specific mechanisms of individual CGs to harness their full potential for targeted therapies.

## 5. Cardiac Glycosides in Physiological and Pathological Processes

**Figure 6:**
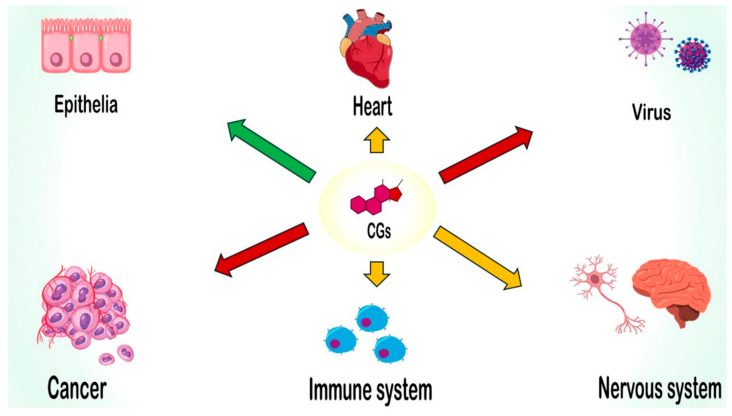
Influence of cardiac glycosides on physiological and pathological processes. Red arrows indicate deleterious effects on target cells. Green arrows represent physiological (beneficial) effects. Yellow arrows indicate context-dependent effects, which may be either beneficial or harmful depending on dosage and target cell type, as discussed in the text.

Initially studied for their cardiotonic and toxic properties, CGs are now recognized for their broader biological relevance. As illustrated in Figure 6, emerging research highlights their involvement in a wide range of physiological and pathological processes with significant biomedical implications.

From a physiological perspective, the discovery that certain CGs are endogenously synthesized has stimulated interest in their diverse biological roles [120]. CGs function as modulators of the epithelial phenotype and play critical roles in regulating blood pressure, maintaining volume homeostasis, and controlling plasma sodium levels.

Pathologically, CGs influence numerous molecular mechanisms implicated in major diseases. Cancer has emerged as a particularly important area, with increasing evidence supporting the impact of CGs on tumor progression and therapeutic response. Additionally, CGs have shown potential as anti-inflammatory and antiviral agents, as well as modulators of immune and nervous system-related disorders—further underscoring their broad therapeutic relevance [20,121,122].

The following subsections provide an updated overview of the roles of various CGs in these key physiological and pathological contexts.

### 5.1. CGs’ Role in Cardiac Function and Regulation

CGs are best known for their effects on cardiac function. Although hundreds of CGs are found in nature, only a few—ouabain, digitoxin, and digoxin—have been used clinically. For nearly two centuries, these compounds were employed to manage heart conditions. However, ouabain and digitoxin were eventually discontinued due to their narrow therapeutic windows and the emergence of safer alternatives, such as beta-blockers and ACE inhibitors [123,124]. Currently, digoxin remains the only CG in clinical use, primarily for treating heart failure with reduced ejection fraction (HFrEF) and atrial fibrillation [125,126].

Digoxin’s relatively short half-life allows for precise dosing and easier toxicity management. Standard dosages range from 0.125 to 0.25 mg/day, with adjustments required in patients with renal impairment [9,127,128]. In overdose scenarios, digoxin-specific antibody fragments (Digibind) are used as an antidote to neutralize circulating digoxin and promote its renal elimination [129].

CGs exert positive inotropic effects by inhibiting NKA in cardiac myocytes. This inhibition raises intracellular sodium, reduces sodium–calcium exchange, and increases intracellular calcium—particularly in the sarcoplasmic reticulum. The resulting calcium surge during depolarization enhances myocardial contractility, improving cardiac output and relieving heart failure symptoms. Additionally, CGs exhibit vagomimetic effects, slowing heart rate and atrioventricular conduction, which is beneficial in atrial fibrillation. Nonetheless, due to their narrow therapeutic index, CGs require careful monitoring to prevent potentially fatal arrhythmias.

### 5.2. CGs as Modulators of Salt (Sodium) and Blood Pressure

Over the past three decades, research has demonstrated that both endogenous and exogenous CGs influence cardiovascular physiology beyond direct cardiac effects, particularly in fluid balance, sodium regulation, and blood pressure control [120,121].

The short-term administration of exogenous CGs—including cardenolides (e.g., ouabain, G-strophanthin, ouabagenin, dihydroouabain) and bufadienolides (e.g., marinobufagenin, telocinobufagin, proscillaridin A)—can enhance cardiac contractility, elevate blood pressure, and promote natriuresis at the renal tubular level [130]. However, long-term exposure to certain CGs is associated with cardiac and renal hypertrophy and the development of hypertension [131]. Interestingly, digoxin does not induce hypertension with chronic use and may even counteract ouabain-induced hypertensive effects [132].

Endogenous cardiac glycosides (ECGs)—such as endogenous ouabain (EO) and marinobufagenin (MBG)—are crucial regulators of blood pressure and sodium balance [133]. Their plasma levels fluctuate with sodium intake, systemic blood pressure, stress, and physical activity. Elevated ECG concentrations are observed in pathological conditions associated with sodium dysregulation, including hypertension [134], hyperaldosteronism [135], renal artery stenosis [136], preeclampsia [45], and heart failure.

ECGs also interact with each other and key systems regulating sodium homeostasis, such as the renin–angiotensin–aldosterone system (RAAS) [137]. EO levels inversely correlate with cardiac function in heart failure [121]. ECGs are implicated in the pathogenesis of several hypertensive conditions [138], including those linked to chronic kidney disease and salt sensitivity [139]. Remarkably, rostafuroxin (PST2238), a digitoxigenin derivative, mitigates EO- and ouabain-induced hypertension.

In summary, ECGs play critical roles in sodium and fluid balance. Elevated sodium in the midbrain stimulates hippocampal ouabain release, triggering ACTH and angiotensin II secretion. These hormones promote adrenal synthesis and the release of ECGs from cholesterol precursors [140].

### 5.3. CGs as Modulators of the Epithelial Phenotype

Epithelia are a primordial structural form in Metazoa, serving as barriers and regulators of solute exchange between compartments. Epithelial cells are characterized by apical-basolateral polarity and specialized junctions, including tight junctions, adherens junctions, desmosomes, and gap junctions [141,142].

The initial observation that ouabain induces the detachment of MDCK cells from substrates and each other led Cereijido and colleagues to hypothesize that CGs modulate epithelial phenotype [143,144]. Subsequent evidence confirms that nanomolar concentrations of ouabain modulate key epithelial structures:

- Tight junctions [145].
- Adherens junctions [146].
- Gap junctions [147,148,149].

Other CGs, including digoxin and marinobufagenin, similarly enhance gap junctional intercellular communication [150].

Beyond junctional effects, CGs influence epithelial polarization. For example, ouabain accelerates primary cilium formation in confluent MDCK cells—a marker of epithelial maturity [151].

CGs also regulate ion channel expression, including voltage-gated potassium channels [152] and TRPV4 channels [153]. Notably, these effects depend on cell–cell contact, suggesting that CG signaling is context-sensitive.

Additionally, ouabain stimulates prostaglandin E_2_ (PGE_2_) production and release, which further enhances gap junction communication [154], reinforcing its role in epithelial connectivity through both direct and indirect mechanisms.

**Figure 7:**
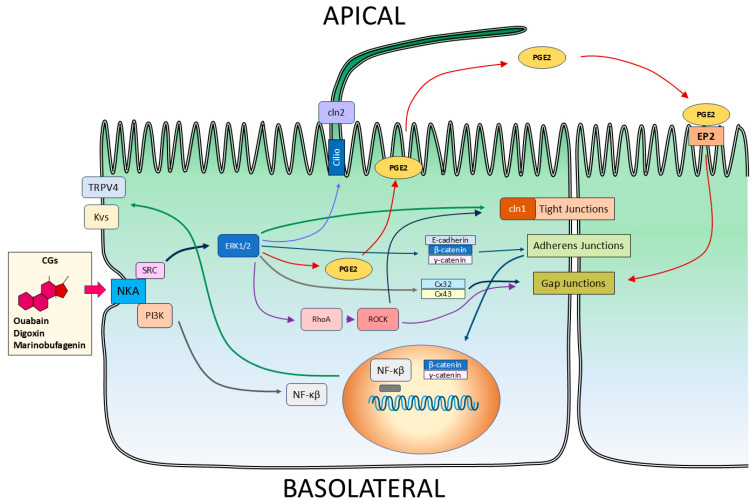
CGs. Influence on key hallmarks of the epithelial phenotype. Color of arrows represent different signalling pathways.

Mechanistically, these effects originate from CG binding to Na^+^/K^+^-ATPase, which functions as both an ion pump and a signal transducer. This interaction activates key intracellular pathways, including c-Src, ERK1/2, PI3K, and Rho/ROCK, thereby orchestrating the phenotypic changes observed in epithelial architecture [155]. Figure 7 schematically illustrates the signaling cascades and epithelial structures modulated by CGs through NKA binding.

### 5.4. CGs’ Influence in Cancer Processes

Cancer comprises a group of over 100 diseases characterized by uncontrolled cell proliferation and the ability to invade surrounding tissues and metastasize to distant organs. While common types include breast, lung, and colorectal cancers, others—such as pancreatic cancer, glioblastoma, and hepatocellular carcinoma—are particularly aggressive and treatment-resistant [156]. Tumor progression involves several hallmark biological capabilities, including self-sufficiency in growth signals, resistance to growth inhibition, the evasion of apoptosis, limitless replicative potential, sustained angiogenesis, and metastatic dissemination [157]. These features result from complex molecular alterations, notably in transcription factors (TFs) that govern inflammation, hypoxia response, proliferation, epithelial–mesenchymal transition (EMT), and cellular plasticity. Key TFs include NF-κB, HIF-1, c-Myc, AP-1, and STAT3 [158].

The potential anticancer activity of cardiac glycosides (CGs) was first suggested by Stenkvist et al. in 1979, who observed reduced tumor aggressiveness in breast cancer patients receiving CG treatment [159]. Since then, numerous studies have reported the anticancer effects of various CGs across diverse malignancies, including breast, lung, pancreatic, colorectal, and liver cancers [160]. These effects encompass multiple cancer hallmarks, including the induction of programmed cell death, the inhibition of metastasis, the suppression of angiogenesis, and the disruption of cell cycle progression. Additionally, CGs modulate epigenetic mechanisms and contribute to overcoming drug resistance, highlighting their potential as multifaceted anticancer agents [161]. Table 1 summarizes recent findings on the anticancer properties of various CGs and their effects on key signaling pathways across different cancer types.

#### 5.4.1. CGs as Inducers of Cancer Cell Death

A defining feature of CG-mediated anticancer activity is their ability to selectively trigger cell death in tumor cells through diverse mechanisms. Below, we review their roles in various forms of programmed cell death, emphasizing recent studies and context-specific effects.

##### CGS as Senolytics

Cellular senescence is a state of stable, irreversible cell cycle arrest triggered by stressors such as DNA damage, oxidative stress, or oncogene activation. Although senescent cells cease proliferating, they remain metabolically active and secrete a pro-inflammatory milieu termed the senescence-associated secretory phenotype (SASP) [199]. In cancer, senescence plays a dual role: it can prevent tumor progression by arresting damaged cells but also promote tumorigenesis by fostering chronic inflammation and remodeling the tumor microenvironment. This paradox has spurred interest in senolytics—agents that selectively eliminate senescent cells—as therapeutic tools in oncology and age-related diseases [200].

Several CGs have emerged as potent senolytic compounds (reviewed in [201]). In a high-throughput screen, Triana-Martínez et al. identified proscillaridin A, ouabain, digoxin, bufalin, cinobufagin, peruvoside, digitoxin, and convallatoxin as promising senolytics [202]. Guerrero et al. further demonstrated that CGs such as ouabain, bufalin, ouabagenin, k-strophanthin, and strophanthidin selectively induce apoptosis in senescent cells via diverse mechanisms [203]. More recently, machine learning-based screens identified periplocin and oleandrin as additional CG-derived senolytics [204]. Resibufogenin, a bufadienolide from toad venom, was shown to eliminate senescent cells via a caspase-3-dependent apoptotic mechanism [205].

##### CGs as Inducers of Apoptosis

Apoptosis is a tightly regulated form of programmed cell death critical for development and tissue homeostasis. It proceeds via two primary pathways: the intrinsic (mitochondrial) pathway, triggered by internal stressors and mediated by cytochrome c, Apaf-1, and caspase-9; and the extrinsic (death receptor) pathway, initiated by ligands such as FasL or TNF-α, leading to caspase-8 activation. Both pathways converge on effector caspases (e.g., caspase-3), which execute cellular dismantling. These pathways are tightly controlled by regulatory proteins, including the Bcl-2 family and inhibitor of apoptosis proteins (IAPs). In cancer, the dysregulation of apoptosis via p53 mutation, Bcl-2 overexpression, or caspase inhibition contributes to tumor persistence and therapy resistance [206,207,208].

Numerous CGs—including digoxin and ouabain—induce apoptosis in cancer cells, primarily by inhibiting NKA pumping activity, which leads to elevated intracellular calcium and reactive oxygen species (ROS) generation. These changes activate both intrinsic and extrinsic apoptotic pathways, disrupt mitochondrial function, modulate apoptotic regulators such as Bcl-2 and p53, and activate caspases. CGs also inhibit pro-survival pathways such as NF-κB, enhancing apoptotic signaling. Importantly, many CGs exhibit cancer-selective toxicity, offering therapeutic advantages [160,209,210]. Table 2 summarizes CG-induced apoptotic mechanisms across various cancer models.

In addition to apoptosis, several non-apoptotic forms of regulated cell death play crucial roles in cancer biology and therapy. Recent studies have highlighted the capacity of cardiac glycosides (CGs) to modulate these pathways—particularly ferroptosis, pyroptosis, and parthanatos—offering novel therapeutic strategies, especially against resistant or heterogeneous tumors.

##### CGs as Inducers of Ferroptosis

Ferroptosis is a distinct form of regulated cell death driven by iron-dependent lipid peroxidation and oxidative damage, characterized by mitochondrial shrinkage and membrane rupture [230,231,232,233]. Key regulators include glutathione peroxidase 4 (GPX4), the cystine/glutamate antiporter system (SLC7A11), and antioxidant signaling via Nrf2. Several bufadienolides—including arenobufagin, bufotalin, bufalin, and resibufogenin—have been shown to induce ferroptosis in cancer cells.

Arenobufagin induces ferroptosis in glioblastoma and gastric cancer by promoting ROS accumulation and suppressing the Nrf2 pathway [163,234,235]. Bufalin causes lipid peroxidation and downregulates the SLC7A11/GPX4 axis in breast cancer [236]. Bufotalin inhibits GPX4 and induces ferroptosis in lung and colorectal cancer via nanoparticle delivery systems [236] Resibufogenin activates ferroptosis in lung cancer through a non-coding RNA-mediated signaling axis involving transferrin receptor regulation [195].

##### CGs as Inducers of Pyroptosis

Pyroptosis is a pro-inflammatory form of regulated cell death characterized by cell swelling, membrane rupture, and the release of cytokines such as interleukin-1β (IL-1β) and IL-18. It is mediated by inflammasome assembly, caspase-1 activation, and gasdermin D (GSDMD) cleavage [206,237]. CGs have been identified as inducers of pyroptosis in cancer and non-cancer contexts. For instance, ouabain activates the NLRP3 inflammasome and induces pyroptosis in cardiomyocytes and immune cells [238]. More recently, bufalin-loaded CaCO_3_ nanoparticles (BCNPs@gel) have been shown to induce Ca^2+^ overload-mediated pyroptosis in tumor models, underscoring the therapeutic potential of CG-based nanomedicines [239].

##### CGs as Inducers of Parthanatos

Parthanatos is a caspase-independent cell death pathway triggered by hyperactivation of poly(ADP-ribose) polymerase-1 (PARP-1), leading to NAD^+^ depletion, mitochondrial dysfunction, and eventual cell death [240]. The synthetic cardenolide ZINC253504760 has been reported to induce parthanatos in leukemia cells [241]. Additionally, hellebrigenin promotes PARP-1-mediated parthanatos in oral squamous cell carcinoma [223], further supporting the role of CGs in engaging this emerging form of regulated cell death.

##### CGs as Inducers of Autophagic Cell Death

Autophagic cell death (ACD) is a regulated form of cell death characterized by excessive autophagy, resulting in the self-digestion and degradation of essential cellular components. Unlike apoptosis, ACD occurs independently of caspase activation and is marked by autophagosome accumulation and lysosomal degradation. It is mediated by autophagy-related proteins such as Beclin-1 and LC3 [242,243]. While physiologically important in development and immunity, ACD plays dual roles in cancer—either suppressing tumor growth or enabling survival under metabolic stress [242,244].

Cardiac glycosides, including cardenolides (e.g., digoxin, digitoxin, ouabain, oleandrin, lanatoside C, Anvirzel and bufadienolides (e.g., bufalin, proscillaridin A), modulate autophagy in a context-dependent manner [210]. They can either stimulate or inhibit autophagy. Inhibition is often associated with autosis, an NKA-dependent form of autophagic cell death. Conversely, CGs can promote autophagy through mechanisms involving AMPK/mTOR and Src/MEK/ERK pathway activation, ROS production, mitochondrial stress, TFEB nuclear translocation, and JNK signaling.

CG-induced autophagy has therapeutic potential, with varying outcomes. In lung cancer, it enhances treatment efficacy, while in gastric and liver cancers, it sensitizes cells to autophagy inhibitors—supporting its value in combinatorial strategies [245].

##### CGs as Inducers of Immunogenic Cell Death

Immunogenic cell death (ICD) is a regulated form of cell death that activates adaptive antitumor immunity, primarily through the stimulation of cytotoxic T lymphocytes. ICD is characterized by the release of damage-associated molecular patterns (DAMPs) such as calreticulin (CRT), ATP, high-mobility group box 1 (HMGB1), and type I interferons, which promote dendritic cell activation and immune cell recruitment [246,247,248]. These features make ICD a promising strategy in cancer immunotherapy [249].

CGs such as digoxin, digitoxin, ouabain, and lanatoside C have been shown to induce the hallmark features of ICD, with immune-stimulating effects comparable to chemotherapeutics like methotrexate [250]. Other CGs, including Scillaren A, proscillaridin, and digitoxigenin, also elicit ICD responses [251]. Notably, digoxin enhances CRT exposure and ATP release when used in combination with cisplatin(IV)–polymer conjugates [252]. In breast cancer models, oleandrin promotes ICD through CRT exposure and the release of HMGB1, HSP70/90, and ATP [118], further supporting the immunomodulatory capacity of CGs.

#### 5.4.2. CGs as Inducers of Cell Cycle (G2/M) Arrest

The G2/M checkpoint ensures that cells do not enter mitosis until DNA replication is complete and genomic integrity is preserved. The dysfunction of this checkpoint is common in cancer and contributes to uncontrolled proliferation. Agents that induce G2/M arrest can exploit this vulnerability to inhibit tumor growth, promote DNA damage responses, and trigger apoptosis [253].

Cardiac glycosides have been shown to induce G2/M arrest in various cancer types:

- Lanatoside C in human prostate cancer cells [184].
- Bufalin in head and neck cancer [254] and lung cancer [255].
- Telocinobufagin in head and neck squamous carcinoma [256], oral squamous carcinoma [223], and HL-60 leukemia cells [257].
- Arenobufagin in A549 lung cancer cells [211].
- ZINC253504760 in leukemia [241]
- Resibufogenin in glioma [258].
- Cinobufagin in colorectal, hepatocellular, and melanoma cells [220].
- Digoxin in lung cancer cells [259].

These findings suggest that CG-induced G2/M arrest is a broadly conserved and therapeutically exploitable mechanism.

#### 5.4.3. CGs as Modulators of Angiogenesis

Hypoxia in tumors—caused by rapid proliferation and poor vascularization—triggers the stabilization of hypoxia-inducible factor 1α (HIF-1α), a transcription factor that induces pro-survival and pro-angiogenic genes, including vascular endothelial growth factor (VEGF) [260,261,262]. Targeting hypoxia and angiogenesis is a major strategy in cancer therapy [263].

Several CGs inhibit angiogenesis by blocking HIF-1α synthesis, even under hypoxic conditions [264]. For example:

- Digitoxin reduces HIF-1α and STAT3 expression, induces apoptosis, and limits proliferation in KRAS-mutant colon cancer cells [222]. It also impairs glioma stemness by downregulating HIF-1α [109].
- Bufalin exerts potent antiangiogenic effects by inhibiting multiple signaling axes, including STAT3, mTOR/HIF-1α, and VEGF, across various malignancies such as ovarian and liver cancers [111,265,266].
- CG derivatives from *Calotropis gigantea* exhibit greater inhibitory effects on HIF-1α than digoxin, indicating the potential for enhanced antiangiogenic efficacy [112].

Moreover, advanced drug delivery systems—such as hypoxia-responsive micelles and dual-loaded nanocarriers—have improved CG efficacy against HIF-1α, augmenting their therapeutic potential [267,268].

#### 5.4.4. CGs as Inhibitors of EMT and Metastasis

Metastasis—the leading cause of cancer mortality—is driven by epithelial–mesenchymal transition (EMT), where epithelial cells acquire mesenchymal features that enhance motility and invasiveness. EMT is mediated by transcription factors like Snail, Slug, and Twist and is associated with poor prognosis and resistance to therapy [269,270].

Multiple CGs have demonstrated anti-EMT activity via diverse mechanisms:

- Arenobufagin suppresses EMT by:
  - Downregulating c-MYC/Nrf2 in colorectal cancer [271].
  - Inhibiting IKKβ/NF-κB in lung cancer [166].
  - Reducing β-catenin levels in prostate cancer [164].
- Bufalin exerts multi-pathway EMT inhibition:
  - Blocking the c-Kit/Slug axis [272].
  - Inhibiting STAT3 signaling [273].
  - Modulating the SRC-3/c-Myc pathway in colorectal cancer [168].
  - Targeting PI3K/AKT/mTOR in gastric cancer [102].
  - Suppressing Src signaling in non-small cell lung cancer [255].
- Bufotalin inhibits EMT through the STAT3/EMT axis in triple-negative breast cancer [217].
- Cinobufagin:
  - Induces Enkurin expression and regulates the β-catenin/c-Jun/MYH9/p53 axis in nasopharyngeal carcinoma [274].
  - Inhibits FAK/STAT3 signaling in triple-negative breast cancer [275].
- Cinobufotalin suppresses EMT in hepatocellular carcinoma by downregulating β-catenin [276].
- Telocinobufagin shows promising EMT-inhibitory activity in undifferentiated thyroid carcinoma, although the specific molecular mechanisms remain to be clarified [196].

Together, these studies highlight the potential of CGs to suppress EMT and limit metastatic spread.

In summary, cardiac glycosides (CGs) have emerged as potent modulators of cancer biology, acting across multiple hallmarks of malignancy. In addition to their classical cardiotonic effects, CGs exhibit anticancer properties through diverse mechanisms, including the induction of apoptosis and alternative forms of regulated cell death (e.g., ferroptosis, pyroptosis, parthanatos, autophagic death, immunogenic death). They also modulate cell cycle progression, suppress angiogenesis, and inhibit EMT and metastasis. Their selective cytotoxicity toward cancer and senescent cells, coupled with their immunomodulatory potential, positions CGs as promising candidates for oncological therapeutics. Future studies should aim to define optimal dosing, delivery strategies, and combination regimens to maximize their efficacy while minimizing toxicity.

### 5.5. Cardiac Glycosides as Immunomodulators: Dual Roles in Inflammation and Adaptive Immunity

Emerging evidence indicates that cardiac glycosides (CGs) possess immunomodulatory properties, acting through a range of molecular targets and signaling pathways that regulate immune cell activation, cytokine production, and inflammatory responses. Among these, digoxin has garnered particular attention due to its interaction with the nuclear receptors retinoic acid receptor-related orphan receptors γ and γt (RORγ and RORγt)—transcription factors central to the differentiation of T helper 17 (Th17) cells and the regulation of pro-inflammatory cytokines. In addition to modulating RORγ signaling, CGs influence key intracellular pathways, including NF-κB, PI3K/Akt, and JAK/STAT, highlighting their potential therapeutic utility in autoimmune diseases, chronic inflammatory disorders, and cancer-associated immune dysregulation [210,277].

#### 5.5.1. Digoxin as an Inhibitor of Th17 Cell Differentiation

Pioneering work by Huh et al. [278] identified digoxin as a selective RORγt antagonist using a Drosophila-based reporter assay. Digoxin inhibited Th17 cell differentiation and suppressed IL-17 transcription without significantly affecting other T cell subsets. In murine models of experimental autoimmune encephalomyelitis (EAE), digoxin treatment delayed disease onset and mitigated neurological symptoms. Furthermore, synthetic derivatives such as 20,22-dihydrodigoxin-21,23-diol and digoxin-21-salicylidene demonstrated low toxicity while selectively inhibiting IL-17 production in human CD4^+^ T cells. Complementary studies by Fujita-Sato et al. confirmed that digoxin directly binds to the ligand-binding domain of RORγt, effectively suppressing Th17 differentiation while sparing Th1 cells and RORα signaling [279].

The anti-inflammatory effects of digoxin have been validated across various disease models:

- Arthritis: Digoxin reduced inflammation and disease progression in both adjuvant-induced [280] and collagen-induced arthritis [281].
- Multiple sclerosis: In EAE models, digoxin attenuated disease severity by inhibiting RORγt and promoting oligodendrocyte differentiation and remyelination. Notably, full remission was achieved when digoxin was combined with tolerance-inducing nanoparticle therapy [282]
- Colitis: Digoxin alleviated colitis symptoms by downregulating IL-17A and IL-17F expression while upregulating the anti-inflammatory cytokine IL-10 in a manner independent of TNF-α signaling [283].
- Rheumatoid arthritis (RA): Digoxin suppressed Th17 differentiation and reduced the production of key inflammatory cytokines (IL-1β, IL-6, IL-17, IL-23) without significantly altering Th1-related markers [284].

#### 5.5.2. Contradictory Findings: CGs as RORγ Agonists

Despite initial findings characterizing CGs as RORγ antagonists, subsequent studies have revealed agonist-like activity under specific conditions, challenging their previously assumed selectivity. Using a HepG2 cell-based reporter system, Karaś et al. identified multiple CGs—including strophanthidin, digoxigenin, and dihydroouabain—as RORγ and RORγt agonists that enhanced IL-17A and IL-17F expression in human Th17 cells [285]. Strikingly, even nanomolar concentrations of digoxin elicited comparable agonist-like responses.

Further expanding on this, Karwaciak et al. tested a panel of 16 CGs (e.g., bufalin, oleandrin, lanatoside C, digitoxin) and observed the upregulation of RORγ target genes such as G6PC, as well as the increased expression of pro-inflammatory cytokines (IL-17A, IL-17F, IFN-γ) and chemokines (CXCL10) in primary human Th17 cells [286]. Supporting the potential pro-inflammatory effects of CGs, a population-based cohort study found a statistically significant association between long-term digoxin use and the increased incidence of psoriasis, particularly among patients with comorbidities such as heart failure, diabetes, and hyperlipidemia [287].

These contrasting observations likely reflect dose-dependent, context-specific, and cell type-dependent effects of CGs, influenced by factors such as immune microenvironment, metabolic state, and the experimental system used.

#### 5.5.3. RORγ-Independent Immunomodulation

Beyond RORγ signaling, several CGs exert immunomodulatory effects through alternative pathways, affecting diverse immune cell populations and inflammatory circuits:

- Periplogenin inhibited the JAK2/3–STAT3 axis, reduced pro-inflammatory cytokine production, and suppressed synoviocyte proliferation and migration in models of rheumatoid arthritis [113].
- Cinobufagin (CBG) enhanced the release of IL-1β and TNF-α, partially through the activation of the PI3K/Akt/mTOR pathway [288].
- Bufalin, identified through systems biology approaches, modulated the expression of immune-related genes including S100B, BIRC5, MMP9, and EGFR, suggesting immunoregulatory roles in breast cancer progression [289].
- Gamabufotalin selectively reduced regulatory T cell (Treg) populations while sparing peripheral blood mononuclear cells, indicating a potential role in enhancing antitumor immune responses [290].
- Convallatoxin (CNT) attenuated vascular inflammation in atherosclerosis by promoting anti-inflammatory M2 macrophage polarization via the PPARγ–Integrin αvβ5 pathway; this effect was reversed by the pharmacological inhibition of PPARγ [291].

These findings reveal that CGs can modulate a broad range of immune responses—including T cell differentiation, cytokine secretion, macrophage activation, and tissue inflammation—independently of RORγ activity.

In summary, CGs exhibit a dualistic and context-dependent immunomodulatory profile, acting through both RORγ-dependent and RORγ-independent mechanisms. While some CGs suppress Th17-driven inflammation by antagonizing RORγt, others may paradoxically enhance pro-inflammatory responses via RORγ activation. Additionally, several CGs influence alternative pathways such as JAK/STAT, PI3K/Akt, and PPARγ, impacting innate and adaptive immunity. These complex and sometimes contradictory effects underscore the need for a nuanced understanding of dosage, chemical structure, target cell type, and disease context when considering CGs for immunotherapeutic applications. Future research should aim to delineate the molecular determinants that govern these divergent immune outcomes, thereby enabling the rational development of CG-based therapies for autoimmune diseases, inflammatory disorders, and cancer immunotherapy.

### 5.6. Cardiac Glycosides as Antiviral Agents

Cardiac glycosides (CGs) have emerged as promising broad-spectrum antiviral agents, exhibiting activity against a wide variety of DNA and RNA viruses. Compounds such as digoxin, lanatoside C, bufalin, ouabain, digitoxin, convallatoxin, proscillaridin A, oleandrin, gitoxin, deslanoside, and k-strophanthidin have demonstrated efficacy against numerous pathogens, including herpes simplex virus type 1 (HSV-1), human cytomegalovirus (HCMV), human immunodeficiency virus (HIV), Zika virus (ZIKV), dengue virus (DENV), influenza A virus (IAV), Ebola virus, severe acute respiratory syndrome coronavirus 2 (SARS-CoV-2), infectious hematopoietic necrosis virus (IHNV), and vaccinia virus [161,292].

#### 5.6.1. Mechanisms of Antiviral Action

The antiviral effects of CGs are primarily mediated through their interaction with NKA, particularly the α1 subunit. The binding of CGs to NKA alters intracellular ion homeostasis—most notably by reducing intracellular K^+^ levels—which disrupts essential processes in the viral life cycle, such as RNA synthesis, protein translation, and post-transcriptional processing. This mechanism has been well-documented in infections caused by RSV and IAV [293].

Beyond ionic disruption, CGs also activate NKA-dependent signaling cascades, including the Src–EGFR–Ras–Raf–MEK–ERK pathway. The activation of this pathway has been shown to block clathrin-mediated endocytosis, a key entry mechanism for RSV, coronaviruses, and other enveloped viruses [294]. In HIV, CGs interfere with the RNA splicing machinery, leading to over-splicing and the subsequent depletion of functional viral mRNAs [295]. In HSV-1 infections, CGs reduce viral egress, resulting in lower extracellular viral titers [295]. Importantly, CGs act post-attachment, primarily affecting intracellular stages of viral replication and assembly [296].

#### 5.6.2. Experimental Evidence Supporting Antiviral Activity

Numerous experimental studies provide strong support for the antiviral potential of CGs:

- Coronaviruses: Digitoxin and ouabain strongly inhibited human coronaviruses HCoV-229E, HCoV-OC43, and SARS-CoV-2 in primary human nasal epithelial cells and lung organoids. The observed antiviral effects were associated with the activation of the MEK and JNK signaling pathways [297].
- Bunyamwera virus: Digoxin inhibited viral replication in Vero cells by reducing viral protein synthesis and altering cell cycle progression. These effects were abolished in cells expressing a digoxin-resistant NKA, confirming the role of NKA inhibition in mediating antiviral activity [298].
- IHNV: Bufalin suppressed both viral attachment and RNA replication in vitro and significantly improved survival and reduced viral burden in infected rainbow trout in vivo. The mechanism was linked to NKA modulation [39].
- HSV-1: Lanatoside C inhibited HSV-1 replication by activating the NRF2 pathway. NRF2 nuclear translocation reduced viral gene expression and preserved nerve fiber integrity in vivo, highlighting NRF2 as a potential therapeutic target [299].
- SARS-CoV-2 (in silico studies): Computational docking studies by Qayed et al. demonstrated that ouabain, digitoxin, digoxin, and proscillaridin bind strongly to key viral targets, including PLpro, Mpro, RNA-dependent RNA polymerase (RdRp), and AAK1. Ouabain was identified as a dual inhibitor of PLpro and Mpro, while digitoxin specifically targeted RdRp [300].
- Zika virus: Ouabain inhibited ZIKV replication in human neural stem and progenitor cells. In a murine model of congenital Zika syndrome, it significantly reduced viral loads in fetal tissues, enhanced neurogenesis, mitigated fetal growth restriction, and decreased levels of pro-inflammatory cytokines [301].

In summary, CGs represent a novel class of host-targeted antiviral agents with diverse and potent effects against both RNA and DNA viruses. Through NKA inhibition, the disruption of ion homeostasis, and the activation of downstream signaling pathways, CGs interfere with viral entry, replication, RNA splicing, and protein translation. Their broad efficacy against multiple viral families—ranging from flaviviruses and herpesviruses to retroviruses and coronaviruses—underscores their therapeutic potential.

Importantly, their ability to modulate host pathways rather than targeting viral components directly may reduce the likelihood of resistance development. Nonetheless, given their narrow therapeutic window, dose-dependent toxicity, and cell-type specificity, further mechanistic studies, structure–activity relationship analyses, and clinical trials are essential to optimize their use and assess safety in antiviral therapy.

### 5.7. Cardiac Glycosides as Neuromodulators: Emerging Roles in the Nervous System

Beyond their well-characterized cardiac and anticancer properties, cardiac glycosides (CGs) are increasingly recognized as modulators of central nervous system (CNS) function. Recent research indicates that CGs can regulate neuroinflammation, synaptic plasticity, and cognitive processes, suggesting their potential for repurposing in neurological and psychiatric disorders.

#### 5.7.1. Alzheimer’s Disease (AD)

In preclinical models of AD, CGs—particularly ouabain and digoxin—have demonstrated neuroprotective effects:

- Ouabain improved cognitive performance in transgenic AD mice by promoting anti-inflammatory microglial polarization through TREM2 upregulation and PI3K/Akt pathway activation [302]. It also reduced tau pathology by activating TFEB and autophagy [303].
- Digoxin enhanced memory and neuronal survival in a rat model of sporadic AD by suppressing TNF-α and restoring choline acetyltransferase (ChAT) activity [304].
- CGs have also been shown to upregulate miR-132, a neuroprotective microRNA typically downregulated in AD. This upregulation was associated with reduced tau expression and the preservation of neuronal integrity [305].

#### 5.7.2. Bipolar Disorder (BD)

The exogenous administration of ouabain in rodent models induced manic- and depression-like behaviors, recapitulating cognitive and affective symptoms characteristic of BD. These effects were accompanied by elevated levels of pro-inflammatory cytokines and impaired BDNF/TrkB signaling, a pathway critical for synaptic plasticity and mood regulation [306,307].

Interestingly, a hypothesized link between COVID-19-related adrenal damage and reduced endogenous ouabain production has been proposed to contribute to BD symptom exacerbation, pointing to a potential neuroendocrine role of CGs in mood regulation [308].

#### 5.7.3. Epilepsy and Multiple Sclerosis

- In a chronic epilepsy (kindling) model, digoxin enhanced the efficacy of sodium valproate, improved seizure control, and reduced markers of neuroinflammation, supporting its use as a potential adjuvant therapy [309].
- In models of demyelination, digoxin promoted oligodendrocyte differentiation and myelin repair, particularly when combined with antigen-specific immune tolerance, showing promise for the treatment of multiple sclerosis (MS) [282].

#### 5.7.4. Synaptic Function and Excitotoxicity

CGs also modulate synaptic signaling and protect against excitotoxic damage:

- Ouabain prevented NMDA-induced excitotoxicity by stabilizing the interaction of Na^+^/K^+^-ATPase (NKA) with NCX and NMDARs within lipid rafts, thereby regulating calcium influx and maintaining synaptic integrity [310].
- Digoxin facilitated dendritic spine formation and improved motor learning, particularly in mice with deficits in the neurotrypsin–agrin signaling pathway [311].

#### 5.7.5. Cognitive Effects in Vascular and Cardiac Contexts

Cognitive benefits of CGs have also been observed in cardiovascular and cerebrovascular disease models:

- In elderly patients with heart failure, digoxin administration was associated with improved cognitive performance [312].
- In a mouse model of chronic cerebral hypoperfusion, digoxin restored glymphatic function and reduced white matter injury, an effect dependent on aquaporin-4 activity [313].

Collectively, these findings highlight the emerging potential of cardiac glycosides as modulators of CNS function. Their ability to influence neuroinflammation, neuroprotection, synaptic signaling, and cognitive outcomes provides a strong rationale for further investigation into their use in neurodegenerative, neuropsychiatric, and neurovascular disorders.

## 6. Cardiac Glycosides Target Molecules Beyond Na^+^/K^+^-ATPase

While the NKA remains the canonical receptor mediating the effects of CGs, growing evidence reveals that CGs can also interact directly with a variety of non-NKA targets, including kinases, membrane receptors, transcription factors, and epigenetic regulators. These interactions highlight the multifaceted mechanisms by which CGs exert their biological effects.

### 6.1. Kinases and Signaling Proteins

Several CGs directly inhibit intracellular kinases that regulate oncogenic and inflammatory pathways:

- CAMKK2 is inhibited by bufalin, leading to the suppression of intrahepatic cholangiocarcinoma via the inhibition of the Wnt/β-catenin pathway [171].
- JAK1, a key mediator of cytokine signaling, is also directly targeted by bufalin, which disrupts the JAK1–ACAP4 interaction, thereby blocking IL-6-induced downstream signaling [314].
- CDK9 and STAT3 have been implicated as targets of acetyl-bufalin in non-small cell lung cancer, contributing to its potent anti-tumor effects [55].

### 6.2. Receptor Tyrosine Kinases

The bufadienolide resibufogenin (RBF) binds directly to the ATP-binding site of VEGFR2, inhibiting its phosphorylation and downstream angiogenic signaling. This impairs endothelial function and suppresses tumor vascularization, especially in triple-negative breast cancer [194].

### 6.3. MAPK Pathway and Transcriptional Regulators

Transcriptomic and molecular docking analyses suggest that CGs can directly modulate MAPK signaling:

- In MCF-7 breast cancer cells, lanatoside C, peruvoside, and strophanthidin were predicted to target MAPK1 and EGR1 [315].
- In hepatocellular carcinoma, bufalin was shown to interact with MAPK1, MAPK14, PRKCA, EIF4E, and APEX1, with binding validated by docking studies and Western blotting [266].

### 6.4. Nuclear Receptors and Transcriptional Regulators

CGs have also been shown to target transcriptional regulators involved in immune and oncogenic signaling:

- CGs bind to the ligand-binding domain of RORγ/RORγT, nuclear receptors involved in immune regulation and tumor progression [286].
- SRC-3, a transcriptional coactivator of c-Myc, is a direct target of bufalin in chemoresistant colorectal cancer. Bufalin-mediated downregulation of SRC-3 suppresses c-Myc expression and metastasis. The overexpression of either SRC-3 or c-Myc reverses these effects, confirming a functional dependency [168].

### 6.5. Other Membrane Receptors

The membrane receptor LRP4 is targeted by both oleandrin and digoxin. Oleandrin inhibits osteoclastogenesis via LRP4 in bone models [104], while digoxin exerts anti-inflammatory and chondroprotective effects through LRP4 in nucleus pulposus cells and osteoarthritis models [316,317].

### 6.6. RNA-Binding Proteins

Telocinobufagin (TBG) binds to LARP1, a translational regulator in the mTOR signaling pathway. TBG disrupts the LARP1–mTOR interaction, leading to the inhibition of metastasis in undifferentiated thyroid cancer [196].

### 6.7. Oncoproteins and Apoptosis Regulators

Bufalin targets the BFAR (Bifunctional Apoptosis Regulator), an anti-apoptotic protein that activates the PI3K/Akt/mTOR pathway. Bufalin binding downregulates the BFAR and suppresses downstream oncogenic signaling in gastric cancer [102].

### 6.8. Epigenetic Regulators

The cardiac glycoside AT2, produced by *Antiaris toxicaria*, directly inhibits UHRF1, a chromatin regulator involved in DNA methylation and gene silencing, thereby revealing a novel NKA-independent mechanism of action [318].

### 6.9. Hypoxia Response Regulators

In a pharmacological screening, HIF-1α was identified as a direct target of digoxin, which inhibited its protein synthesis and transcriptional activity. The anti-tumor effects of digoxin were reversed in cells overexpressing HIF-1α, confirming functional relevance [108].

In summary, beyond NKA, cardiac glycosides interact with a broad spectrum of molecular targets, including kinases, nuclear receptors, RNA-binding proteins, and epigenetic regulators. These diverse interactions significantly contribute to the anticancer, anti-inflammatory, and immunomodulatory effects of CGs, reinforcing their potential for therapeutic repurposing across multiple disease contexts.

## 7. Discussion

Cardiac glycosides (CGs), originally developed for the treatment of heart failure and arrhythmias, have garnered renewed interest as potential therapeutic agents across a wide spectrum of non-cardiac diseases, including cancer, viral infections, autoimmune disorders, and neurodegenerative conditions. This paradigm shift is supported by growing evidence that CGs modulate diverse cellular pathways, extending far beyond their classical role as Na^+^/K^+^-ATPase (NKA) inhibitors.

Although the inhibition of NKA remains a fundamental mechanism underlying the effects of CGs, recent studies have uncovered their capacity to modulate multiple intracellular signaling pathways, including NF-κB, MAPK, PI3K/Akt, JAK/STAT, and mTOR. These pathways regulate key cellular processes such as inflammation, proliferation, apoptosis, and immune responses. Importantly, CGs have been shown to trigger a range of regulated cell death modalities—including apoptosis, ferroptosis, pyroptosis, parthanatos, autophagic cell death, and immunogenic cell death—which may be exploited to overcome therapy resistance in refractory malignancies.

Beyond their well-characterized role in modulating NKA, CGs have been shown to directly engage a wide variety of non-NKA molecular targets. These include receptor tyrosine kinases (e.g., VEGFR2), transcriptional regulators (e.g., SRC-3, RORγ), epigenetic enzymes (e.g., UHRF1), and RNA-binding proteins (e.g., LARP1). These alternative interactions help to explain the context-dependent effects of CGs across different tissues and disease models, broadening their mechanistic and therapeutic repertoire.

In the field of virology, CGs interfere with multiple stages of the viral life cycle—including entry, replication, and RNA processing—particularly in infections caused by coronaviruses, herpesviruses, HIV, and flaviviruses. These antiviral effects are primarily mediated through the disruption of host ion homeostasis and the inhibition of key signaling pathways such as Src–EGFR–Ras–MEK–ERK, highlighting their promise as host-targeted antiviral agents with a lower likelihood of inducing viral resistance.

In immunology, CGs—most notably digoxin—exert immunomodulatory effects by inhibiting RORγt, a nuclear receptor crucial for Th17 cell differentiation. These effects have demonstrated therapeutic potential in models of autoimmune encephalomyelitis, arthritis, and colitis. However, contrasting findings have also emerged: some CGs, or digoxin at specific concentrations or in certain cellular contexts, have shown agonistic activity on RORγ, promoting IL-17 expression. These context- and structure-specific dualities emphasize the complexity of CG pharmacology and underscore the need for further mechanistic clarification.

From a neurological perspective, CGs display neuroprotective properties in preclinical models of Alzheimer’s disease, epilepsy, multiple sclerosis, and vascular cognitive impairment. These effects are attributed to their ability to suppress neuroinflammation, enhance autophagy, regulate calcium signaling, and promote synaptic plasticity. Moreover, CGs have been implicated in mood disorders such as bipolar disorder, with both therapeutic benefits and pathophysiological contributions suggested, depending on endogenous or exogenous exposure.

Despite this broad therapeutic promise, the narrow therapeutic index of CGs—characterized by a fine line between effective and toxic doses—remains a major challenge to clinical translation. Cardiotoxicity, in particular, limits their systemic use and necessitates careful dosing and monitoring.

To overcome these limitations, two principal strategies are under active investigation:

- Medicinal chemistry approaches to develop CG derivatives with reduced toxicity and enhanced selectivity.
- Advanced delivery systems, such as nanoparticles, micelles, and conjugates, which enable tissue-specific delivery and reduce systemic exposure.

Together, these innovations seek to expand the therapeutic window of CGs, facilitating their repurposing as viable treatments for complex modern diseases such as cancer, autoimmune conditions, and neurodegeneration.

## 8. Conclusions

Cardiac glycosides (CGs), long regarded solely as cardiotonic agents, have emerged as a diverse class of pharmacological modulators with compelling potential across oncology, virology, immunology, and neurology. Their capacity to engage both canonical targets—such as Na^+^/K^+^-ATPase—and non-canonical effectors, including kinases, nuclear receptors, transcriptional co-regulators, and epigenetic modulators, situates CGs at the crossroads of multiple therapeutic domains.

As the landscape of CG research evolves, key challenges must be systematically addressed. These include improving target selectivity, enhancing therapeutic safety, and developing advanced delivery platforms capable of minimizing off-target toxicity. In parallel, a deeper mechanistic understanding of CG actions—particularly their context-dependent effects and interactions with cellular signaling networks—remains essential for their rational clinical repurposing.

Moving forward, a multidisciplinary strategy that integrates systems biology, structural pharmacology, and nanotechnology will be vital to fully exploit the therapeutic potential of this ancient yet highly adaptable class of compounds. With continued innovation and rigorous investigation, cardiac glycosides may be repositioned as modern therapeutics for some of the most pressing and complex diseases of our time.

## Figures and Tables

**Table 1:** Diversity of CGs’ impact on cancer types.

Name	Target/Pathway	Effect	Tissue	Ref
Arenobufagin	c-Jun N-terminal kinases (JNK)	Apoptosis	Nasopharyngeal carcinoma	[162]
	MiR-149-5p/AEBP1	Ferroptosis	Glioblastoma	[163]
	β-catenin	EMT	Prostate	[164]
	p62-Keap1-Nrf2	Autophagy	Liver	[165]
	IKKβ/NFκB	Migration	Lung	[166]
Acetyl-bufalin	CDK9/STAT3	Growth	Non-small lung	[55]
Acetyl-cinobufagin	STAT3	Proliferation, migration, EMT	Breast	[167]
Bufalin	SRC-3/c-Myc	Metastasis	Colon	[168]
	SRC-3/HIF-1α	Glycolysis	Colon	[169]
	Ca^2+^/CaMKKβ/AMPK/Beclin1	Apoptosis, autophagy	Osteosarcoma	[170]
	CAMKK2/Wnt/β-catenin	Proliferation, metastasis	Bile ducts	[171]
	Hippo-YAP	Proliferation	Lung	[172]
	PIAS3/STAT3	Proliferation, migration, invasion	Esophagus	[173]
	BFAR/PI3K/AKT/mTOR	Metastasis	Stomach	[102]
	AK/STAT, Wnt/β-Catenin, mTOR, TRAIL/TRAIL-R	Proliferation, metastasis	Various	[174]
Bufotalin	AKT	Apoptosis	Glioblastoma	[175]
Cerberin	PI3K/AKT/mTOR	Apoptosis	ND	[101]
Cinobufagin	PI3K/AKT, MAPK/ERK	Growth	Lung	[176]
Cinobufotalin	USP36/c-Myc axis	Proliferation, migration, invasion	Colon	[177]
Convallatoxin	Wnt/β-catenin	Proliferation, migration, invasion	Bone	[178]
Digitoxin	NF-κB/ST6GAL1	Proliferation, migration	Liver	[179]
Digoxin	HIF-1α	Growth	ND	[108]
	STAT3	Migration	Lung	[180]
Gamabufotalin	TGF-β/periostin/PI3K/AKT	Metastasis	Bones	[181]
	NAK(ATP1A3)-AQP4	Growth	Glioblastoma	[182]
Lanatoside C	MAPK, Wnt, JAK-STAT, and PI3K/AKT/mTOR	Growth	Breast, lung, liver	[183]
	TNF/IL-17	Proliferation, apoptosis	Prostate	[184]
Malayoside	MAPK-Nur77	Apoptosis	Non-small lung	[185]
Odoroside A	ROS/JNK	Proliferation	Leukemia	[186]
	STAT-3	Invasion	Breast	[187]
Oleandrin	PERK/elF2α/ATF4/CHOP	Immunogenic cell death	Breast	[118,188]
Ouabain	AMPK-Src	Autophagy, metabolism		[189]
Peruvoside	MAPK Wnt/β-catenin, PI3K/AKT/mTOR	Growth	Breast, lung, and liver	[117]
	Src-EGFR	Growth, invasion	Lung	[190]
Periplogenin	JAK2/3-STAT3	Growth	Esophagus	[191]
Resibufogenin	PI3K/AKT a	Growth, migration	Ovary	[192]
Resibufogenin	PI3K/Akt	Viability, migration invasion,	Bone marrow	[193]
	VEGFR2-(VEG)	Angiogenesis	Breast	[194]
	lncRNA LINC00597/hsa-miR-367-3p/TFRC	Ferroptosis	Lung	[195]
Telocinobufagin	LARP1-mTOR	Metastasis	Thyroid	[196]
	STAT3/PARP1	Apoptosis	Lung	[197]
αldiginoside	JAK-STAT	Apoptosis	ND	[198]

**Table 2:** Variety of CGs inducing apoptosis in cancer cells through distinct targets and signaling mechanisms.

CG Name	Target or Pathway	Cancer Type	Ref
21-Benzylidene digoxin	(−) EGFR/ERK	HeLa cells	[50]
Arenobufagin	Modulating claspin and JNK pathway	Nasopharyngeal carcinoma cells	[162]
	Induces apoptosis and G2/M arrest	A549 cells	[211]
	(−) PI3K/AKT/mTOR	Pancreatic Cancer Cells	[212]
Bufalin	Ca^2+^/CaMKKβ/AMPK/Beclin1	Osteosarcoma cells	[170]
	Annexin A2 and DRP1 regulation	Glioma cells	[213]
	ROS	Neuroblastoma	[214]
	Unspecified	Glioma	[215]
Bufarenogin	Bax and ANT cooperation	Unspecified	[216]
Bufotalin	Mitochondrial dysfunction via AKT signaling pathway	Glioblastoma cells	[175]
	Inhibiting the STAT3/EMT Axis	Triple-negative breast cancer cells	[217]
Cinobufagin	G9a	Non-small-cell lung cancer A549 cells	[218]
	(−) β-catenin signaling	Acute promyelocytic leukemia	[219]
	DNA damage response, G2/M checkpoint	Unspecified cancer cells	[220]
	Unspecified	Nasopharyngeal carcinoma cells	[221]
Convallatoxin	JAK2/STAT3 and mTOR/STAT3	Colorectal cancer	[116]
Digitoxin	HIF-1α and STAT3	KRAS mutant human colon cancer cells	[222]
Hellebrigenin	MAPK signaling and XIAP expression	Oral cancer	[223]
Lanatoside C	TNF/IL-17 signaling pathway	Human prostate cancer cells	[184]
	Inhibition of STAT3	Cholangiocarcinoma	[224]
Malayoside	MAPK-Nur77 signaling	Human non-small lung cancer cells	[185]
Oleandrin	ROS-ER Stress	Breast cancer cells	[225]
Ouabain	Induction of apoptosis, G2/M arrest, migration inhibition	Melanoma cells	[226]
Periplocymarin	PI3K/AKT pathway	Colorectal cancer cells	[227]
Periplogenin	ROS-ER stress	Unspecified	[228]
Peruvoside	MAPK, Wnt/β-catenin, PI3K/AKT/mTOR	Human cancers	[117]
Strophanthidin	Promoting TRAIL-DR5 signaling	Lung Adenocarcinoma	[229]

## Abbreviations

The following abbreviations are used in this manuscript:

ACTH	Adrenocorticotropic hormone
ACD	Autophagic cell death
AMOG	Adhesion Molecule on Glia
AP-1	Activator protein 1
ATP	Adenosine triphosphate
CAMK	Calcium/calmodulin-dependent protein kinase
CAFs	Cancer-associated fibroblasts
CaMKKβ	Calcium/calmodulin-dependent protein kinase kinase β
CGs	Cardiac glycosides
CRT	Calreticulin
DAMPs	Damage-associated molecular patterns
EGFR	Epidermal growth factor receptor
EMT	Epithelial–mesenchymal transition
EO	Endogenous ouabain
ER	Endoplasmic reticulum
ERK	Extracellular signal-regulated kinase
FXYD	Single-span transmembrane regulatory protein family of NKA
GPX4	Glutathione peroxidase 4
GSH	Glutathione
HCMV	Human cytomegalovirus
HIF-1α	Hypoxia-inducible factor 1 alpha
HMGB1	High mobility group box 1
HSV	Herpes simplex virus
ICD	Immunogenic cell death
IFN-γ	Interferon gamma
IL	Interleukin
IP3R	Inositol 1,4,5-trisphosphate receptor
JAK	Janus kinase
MAPK	Mitogen-activated protein kinase
mTOR	Mammalian target of rapamycin
NKA	Na^+^/K^+^-ATPase
NF-κB	Nuclear factor kappa-light-chain-enhancer of activated B cells
NLRP3	NOD-, LRR-, and pyrin domain-containing protein 3
NRF2	Nuclear factor erythroid 2–related factor 2
PARP-1	Poly(ADP-ribose) polymerase 1
PERK	PKR-like ER kinase
PI3K	Phosphoinositide 3-kinase
PKC	Protein kinase C
PLC	Phospholipase C
RORγ	Retinoic acid-related orphan receptor gamma
ROS	Reactive oxygen species
SASP	Senescence-associated secretory phenotype
Src	Proto-oncogene tyrosine-protein kinase Src
STAT	Signal transducer and activator of transcription
TFEB	Transcription factor EB
Th17	T helper 17 cells
TGF-β	Transforming growth factor beta
TNF-α	Tumor necrosis factor alpha
TRAIL	TNF-related apoptosis-inducing ligand
UPR	Unfolded protein response
VEGF	Vascular endothelial growth factor
